# Supplementary material for: Temporal dynamics of antigen-specific T cell expansion in primary SARS-CoV-2 infection
Source: Sci Adv. 2026 Nov 6;12(45):eaec4195. doi: 10.1126/sciadv.aec4195 (PMC13742180; doi:10.1126/sciadv.aec4195)
Supplement: Supplementary file 1 [file sciadv.aec4195_sm.pdf]

Science Advances
AAAS

Supplementary Materials for

Temporal dynamics of antigen-specific T cell expansion in primary SARS-CoV-2 infection

Andrew G. T. Pyo *et al.*

Corresponding author: Andrew G. T. Pyo, agpyo@stanford.edu; Ned S. Wingreen, wingreen@princeton.edu; Benny Chain, b.chain@ucl.ac.uk

Sci. Adv. **12**, eaec4195 (2026)
DOI: 10.1126/sciadv.aec4195

This PDF file includes:

Supplementary Text
Tables S1 and S2
Figs. S1 to S9
References

1 Mathematical model

In this section, we outline the model, which closely follows the framework developed by de Boer and Perelson [13].

We consider a scheme where, in a given lymph node, a naive T cell of clonotype i, denoted $\mathcal{N}_i$, interacts with a peptide-loaded binding site on antigen-presenting cells (APCs), denoted $\mathcal{S}$, to form an interaction complex $\mathcal{C}_i$, with association and dissociation rates k_i^+ and k_i^-, respectively. Upon complex formation, naive T cells can be activated at rate k_i^*, becoming an activated T cell, denoted $\mathcal{T}_i^*$. This process is summarized by:

$$\mathcal{N}_i + \mathcal{S} \underset{k_i^-}{\overset{k_i^+}{\rightleftarrows}} \mathcal{C}_i \xrightarrow{k_i^*} \mathcal{T}_i^* + \mathcal{S}. \tag{S1}$$

We denote the total naive T cell population in clonotype i (including both free T cells $\mathcal{N}_i$ and complexes $\mathcal{C}_i$) by N_i. Its dynamics are:

$$\dot{N}_i = J_i - k_i^* C_i, \tag{S2}$$

where J_i denotes the influx of naive T cells, and C_i is the number of complexes. The activated T cell population, denoted T_i^*, evolves as:

$$\dot{T}_i^* = k_i^* C_i. \tag{S3}$$

Activated T cells proliferate and differentiate into effector T cells T_i, which medicate clearance of virus-infected targets; we model this contribution using a law-of-mass action term [15, 16]:

$$\dot{V} = \alpha_V V \left(1 - \frac{V}{V_\infty}\right) - \gamma_V V \sum_j T_j, \tag{S4}$$

where V is the viral load, α_V is the viral replication rate, and γ_V is the rate of virus elimination by T cells.

To model antigen presentation, let $\mathcal{S}_{\text{free}}$ represent unoccupied peptide-presentation sites in a given lymph node. Presentation of viral particles $\mathcal{V}$ to these free sites proceeds via

$$\mathcal{V} + \mathcal{S}_{\text{free}} \underset{a^-}{\overset{a^+}{\rightleftarrows}} \mathcal{S}, \tag{S5}$$

where a^+ is the rate of antigen presentation, and a^- is the decay rate of presented antigen.

Within a given lymph node, let S_{tot} denote the total number of potential peptide-presentation sites, and define $S_{\text{free}} = S_{\text{tot}} - S$ as the number of unoccupied sites. Here, S denotes the total number of sites currently presenting peptide, including both unbound antigen-presenting sites $\mathcal{S}$ and those bound to T cells of any clonotype (i.e. $\sum_i C_i$). The dynamics of antigen presentation is then given by

$$\dot{S} = a^+(S_{\text{tot}} - S)V - a^- S. \tag{S6}$$

1.1 Quasi-steady state approximations

1.1.1 Antigen presentation

Assuming quasi-steady state ($\dot{S} = 0$), the total number of peptide-loaded sites becomes:

$$S = S_{\text{tot}} \frac{V}{\kappa + V}, \tag{S7}$$

where $\kappa \equiv a^-/a^+$ represents the inverse of antigen presentation efficiency.

1.1.2 Interaction complexes

From above framework, the dynamics of C_i can be shown to follow [5, 14, 37]:

$$\dot{C}_i = k_i^+ \left(S - \sum_j C_j \right) (N_i - C_i) - (k_i^- + k_i^*) C_i. \tag{S8}$$

Assuming a quasi-steady state for each C_i, and expanding to first order in C_i, i.e. $\sim \mathcal{O}(C_i)$ [38], we obtain:

$$C_i = \frac{S N_i}{(S + K_i)\left(1 + \sum_j \frac{N_j}{S + K_j}\right)}, \tag{S9}$$

where $1/K_i \equiv k_i^+/(k_i^- + k_i^*)$ characterizes the affinity of T cells for the antigen.

1.1.3 Naive T cell balance

To simplify, we assume that influx exactly balances activation ($J_i = k_i^* C_i$), so the precursor pool remains constant. This gives:

$$J_i = k_i^* C_i \quad \forall i. \tag{S10}$$

Thus, the activation rate depends on the viral load via $S(V)$:

$$r_i(V) = \frac{k_i^* S(V) N_i}{[S(V) + K_i]\left[1 + \sum_j \frac{N_j}{S(V) + K_j}\right]}. \tag{S11}$$

1.2 Stochastic activation via inhomogeneous Poisson process

We model activation of naive T cells as a discrete stochastic process in continuous time. For each clonotype i, activation events occur at random times $\{t_{i,n}^*\}_n$, where each event corresponds to the transition of a naive T cell into an activated state. We assume that these activation times are generated by an inhomogeneous Poisson process with a time-dependent rate $r_i(t)$, which depends on antigen availability and TCR affinity.

1.2.1 Definition of activation indicator

Let $r_i(t)$ denote the instantaneous activation rate for clone i. The probability that an activation occurs in the infinitesimal time interval $[t, t + dt)$ is given by

$$\Pr\left[\text{activation in } [t, t + dt)\right] = r_i[V(t)]\, dt. \tag{S12}$$

The corresponding activation indicator function is defined as

$$\xi_i(t) = \sum_n \delta(t - t_{i,n}^*), \tag{S13}$$

where $\delta(\cdot)$ is the Dirac delta function, and $\{t_{i,n}^*\}_n$ are the (random) activation times for clone i. Each term $\delta(t - t_{i,n}^*)$ represents a discrete activation event occurring at time $t_{i,n}^*$.

1.2.2 Effector T cell dynamics and expectations

Following activation, a T cell undergoes deterministic proliferation governed by a response function $h(t, K_i)$ that depends on the time since activation and the affinity $1/K_i$. The effector population for clone i then evolves as:

$$T_i(t) = \int_0^t d\tau\, \xi_i(\tau)\, h(t - \tau, K_i) = \sum_{k=1}^{n_i} h(t - t_{i,k}^*, K_i), \tag{S14}$$

where n_i is the number of activation events of naive T cells of clonotype i. The above expression sums the contributions from all past activation events.

Taking the expectation over realizations of the stochastic process, and using the fact that $\mathbb{E}[\xi_i(t)] = r_i(t)$, we obtain the expected rate of effector accumulation:

$$\mathbb{E}[T_i(t)] = \int_0^t d\tau \, r_i(\tau) \, h(t - \tau, K_i). \tag{S15}$$

It also follows that $\mathbb{E}[n_i] = \int_0^t d\tau \; r_i(\tau)$.

1.2.3 Deterministic proliferation

Upon activation, we assume that the activated T cell proliferates at a fixed rate, γ_e, for an affinity-dependent duration $\mathcal{T}(K_i)$ and then the resulting lineage decays at a fixed rate γ_c. The corresponding response function is

$$h(t, K_i) = \begin{cases} 0, & t < 0 \\ e^{\gamma_\mathrm{e} t}, & 0 \leq t < \mathcal{T}(K_i) \\ e^{\gamma_\mathrm{e} \mathcal{T}(K_i)} e^{-\gamma_\mathrm{c}[t - \mathcal{T}(K_i)]}, & t \geq \mathcal{T}(K_i) \end{cases}. \tag{S16}$$

A simple choice for the duration of proliferation is

$$\mathcal{T}(K_i) = \mathcal{T}_0 \frac{K_0}{K_i}, \tag{S17}$$

where $\mathcal{T}_0$ is a typical duration of proliferation for T cell with affinity $1/K_0$.

1.3 Proliferation limited by antigen-availability

While a fully autonomous division-destiny program is well established for CD8$^+$ T cells, experimental studies indicate that CD4$^+$ T cell proliferation is more sensitive to antigen persistence, particularly during later stages of expansion [25–30]. To assess whether such antigen-dependent maintenance of proliferation could alter our conclusions, we extended the clonal expansion model to allow the duration of proliferation to depend explicitly on antigen availability.

In this formulation, each activated T cell is assigned a nominal proliferative duration $\mathcal{T}$ at the time of activation (Eq. S17), which is subsequently modulated by the instantaneous level of antigen presentation. Specifically, we model the effective proliferative duration as

$$\mathcal{T}(t; K_i) = \mathcal{T}(K_i) \left[1 - 0.25 e^{-15 s(t)}\right], \tag{S18}$$

where $s(t) = S(t)/S_\mathrm{tot}$ denotes the antigen availability scaled by its maximal value. The choice allows proliferation to terminate prematurely when antigen becomes limiting, with a maximal reduction of 25% relative to the nominal division program, while preserving near-autonomous expansion under high-antigen conditions.

This extension captures a regime in which early clonal expansion is partially programmed but can be curtailed by declining antigen levels, consistent with experimental observations of CD4$^+$ T cell responses. Importantly, incorporating antigen-dependent limitation of proliferation does not qualitatively alter the relationship between peak timing and peak magnitude observed in the model, As shown in Fig. S6, variation in precursor frequency continues to produce little or no correlation between peak day and peak abundance, whereas affinity-driven variations robustly induces a positive correlation. Thus, the central conclusions of the model are robust to whether post-activation proliferation is fully autonomous of modulated by ongoing antigen availability.

2 Parameters

2.1 Number of naive T cells

There are $\sim 10^{11}$ naive T cells in humans [39], where a fraction of $10^{-6} - 10^{-5}$ are specific to any given antigen [40]. Given that there are $\sim 10^8$ unique clonotypes of naive T cells in humans [2,41], this implies that for a particular antigen there are $N_{\text{clone}} \sim 10^2 - 10^3$ specific T cell clones, with average $\sim 10^3$ naive T cells per clone.

The average number of T cells in the same clonotype that is present in a specific LN can be estimated as

$$N_0 \approx \frac{\text{Time spent in LN}}{\text{Time spent in LN} + \text{Time between LNs}} \times \frac{\#\text{ T cells per clone}}{\#\text{ LNs}} \sim \mathcal{O}(10) \text{ cells/LN.} \quad \text{(S19)}$$

2.1.1 Interaction kinetics

Assuming that at most $\sim 10\%$ of naive T cells are interacting with a presented antigen at any given time (i.e., in the limit $V \gg \kappa$), we use Eq. S9 to estimate the typical number of interacting cells:

$$\frac{C_0}{N_0} \approx \frac{S_{\text{tot}}}{(S_{\text{tot}} + K_0)\left(1 + \frac{N_{\text{clone}} N_0}{S_{\text{tot}} + K_0}\right)} = 0.1. \quad \text{(S20)}$$

Solving for $1/K_0$ yields

$$1/K_0 \approx 10^{-5} \text{ per cell}, \quad \text{(S21)}$$

which is consistent with the estimates of de Boer and Perelson [13].

Similarly, DC-T cell interaction typically lasts 3–4 minutes [42]. Assuming productive interactions are rare, such that $k_i^*/k_i^- \approx 0.01$ corresponding to approximately 1% of DC-T cell interactions leading to activation [13], we have that

$$k_i^- \sim \mathcal{O}(10) \text{ h}^{-1} \quad \text{and} \quad k_i^* \sim \mathcal{O}(10^{-1}) \text{ h}^{-1}. \quad \text{(S22)}$$

3 Figures and tables

Participant #	Chain	Unique clones	Recurrent clones
1	alpha	315303	37856
1	beta	514709	35655
2	alpha	640065	71489
2	beta	1069548	48289
3	alpha	557278	68559
3	beta	934900	57782
4	alpha	612845	91285
4	beta	974290	78784
5	alpha	424371	51973
5	beta	688793	44398
6	alpha	558045	84366
6	beta	905348	81552
7	alpha	502879	59522
7	beta	812491	43188
8	alpha	521439	71565
8	beta	891977	63348
9	alpha	533276	69703
9	beta	938905	60561
10	alpha	620408	80872
10	beta	1008828	63856

Table S1. Number of unique T cell clones. Unique T cell clones were identified at the nucleotide level from the TCR alpha or beta chain across the 11 sampled timepoints. Unique clones correspond to clones detected at least once across all timepoints. Recurrent clones refer to clones detected in at least two distinct timepoints.

Parameter	Description	Value	Reference
γ_e	T cell proliferation rate	$1/8\ \mathrm{h}^{-1}$	Current work
γ_c	T cell decay rate	$1/56.5\ \mathrm{h}^{-1}$	Current work
$\mathcal{T}_0$	Proliferation duration	$2-3$ d	Marchingo et al. Science 2014 [14]
$1/K_i$	T cell affinity parameter	10^{-5} per cell	See SI
k_i^*	T cell activation rate	$10^{-1}\ \mathrm{h}^{-1}$	See SI
N_clone	Number of antigen-specific clones	10^2-10^3	See SI
S_tot	Number of binding sites on antigen-presenting cells in a LN	10^4	de Boer & Perelson J. theor. Biol. 1995 [13]
κ	Antigen presentation saturation constant in a LN	0.1	de Boer & Perelson J. theor. Biol. 1995 [13]
α_V	Viral replication rate	$10^{-1}\ \mathrm{h}^{-1}$	N/A
γ_V	Virus elimination rate	$10^{-5}\ \mathrm{h}^{-1}$	N/A

Table S2. Simulation parameters.

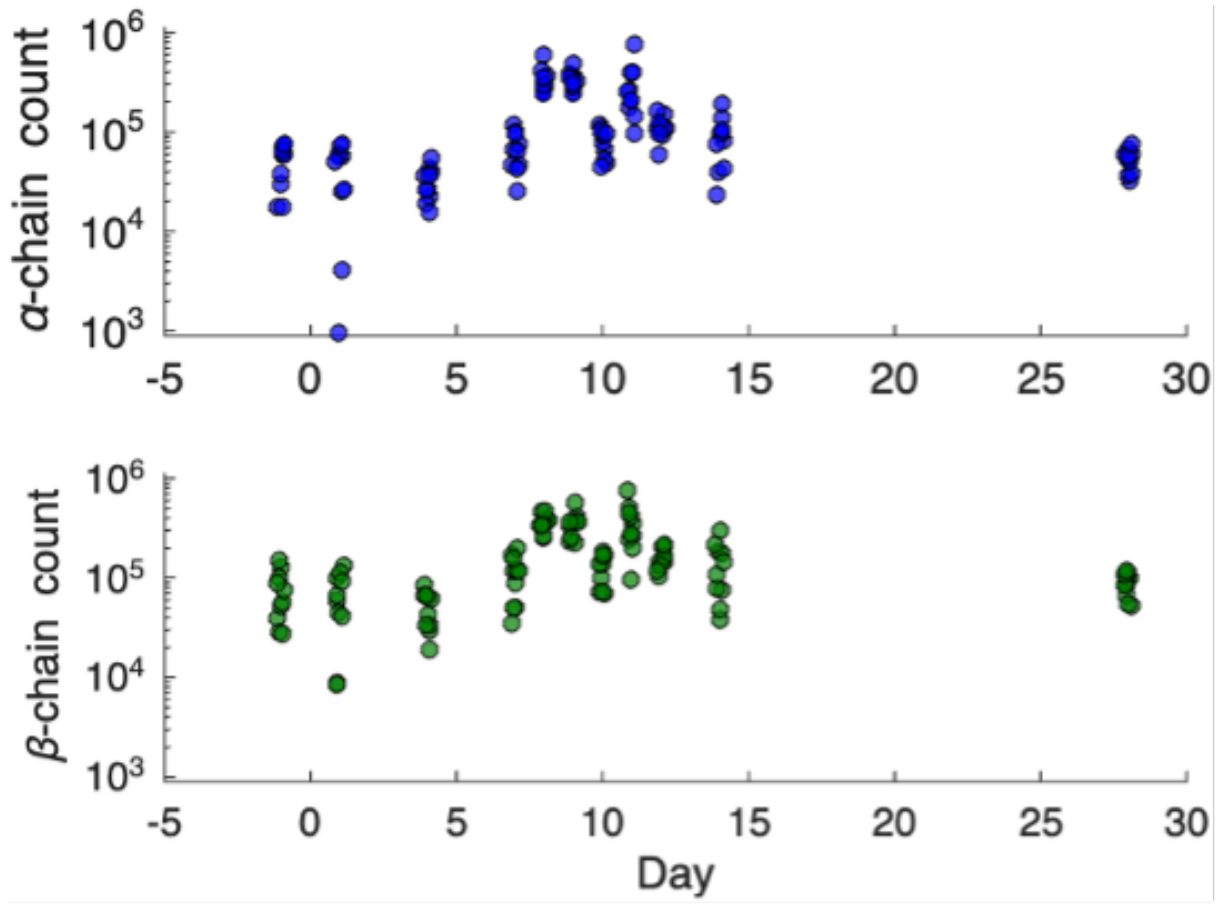

Figure S1. Identification and characterization of expanding T cell clones. Total number of TCR sequencing reads per individual per day. The number of bulk TCR sequencing reads for the α-chain (top) and β-chain (bottom) is shown. Each dot represents the total number of reads obtained from one individual on a given day, across the 10 individuals analyzed.

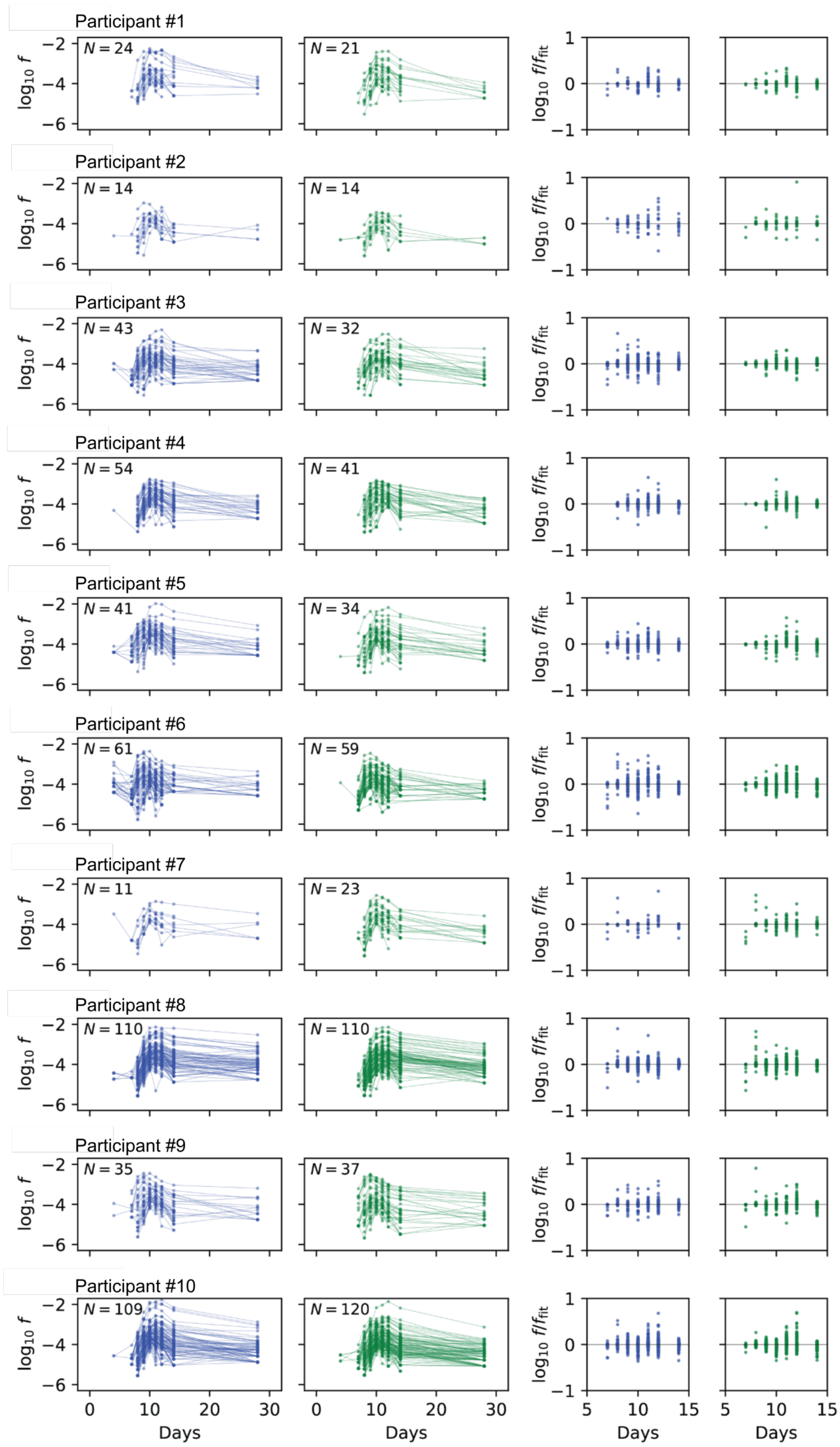

Figure S2. Time course of abundance of expanding T cell clones. Each row represents an individual patient analyzed in the study. The time courses of α-chain and β-chain abundances are shown in the first and second columns, respectively. The residuals between the raw abundances and the fitted trajectories are shown in the third and fourth columns, with α-chain residuals in the third column and β-chain residuals in the fourth column.

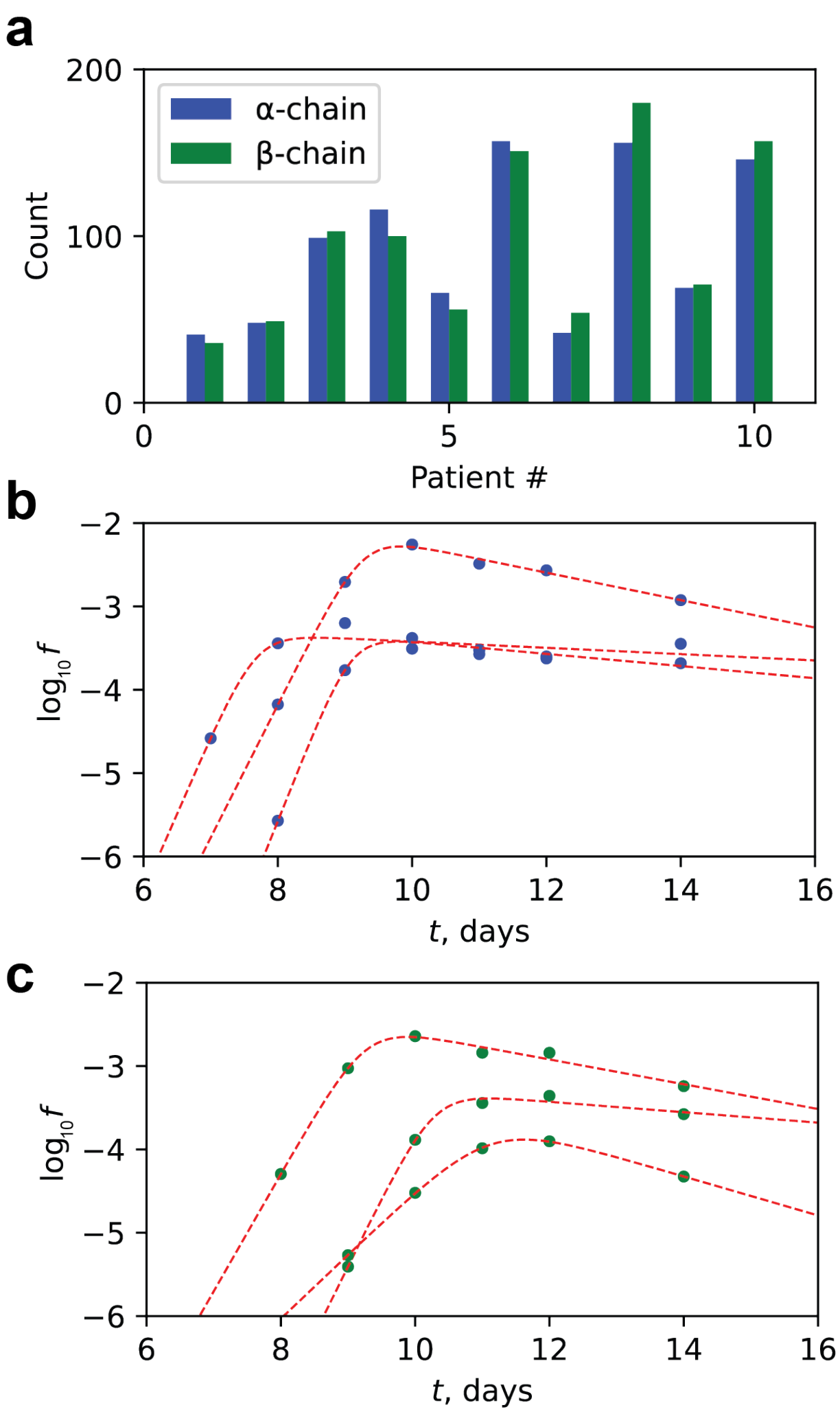

Figure S3. Identification and characterization of expanding T cell clones. a, Number of expanding clones identified in each individual. **b,c,** Examples of longitudinal clonal abundance data constructed between day 7 and day 14 for representative α-chain (**b**) and β-chain (**c**) TCR clones. Solid dots represent observed data points; dotted lines indicate model fits.

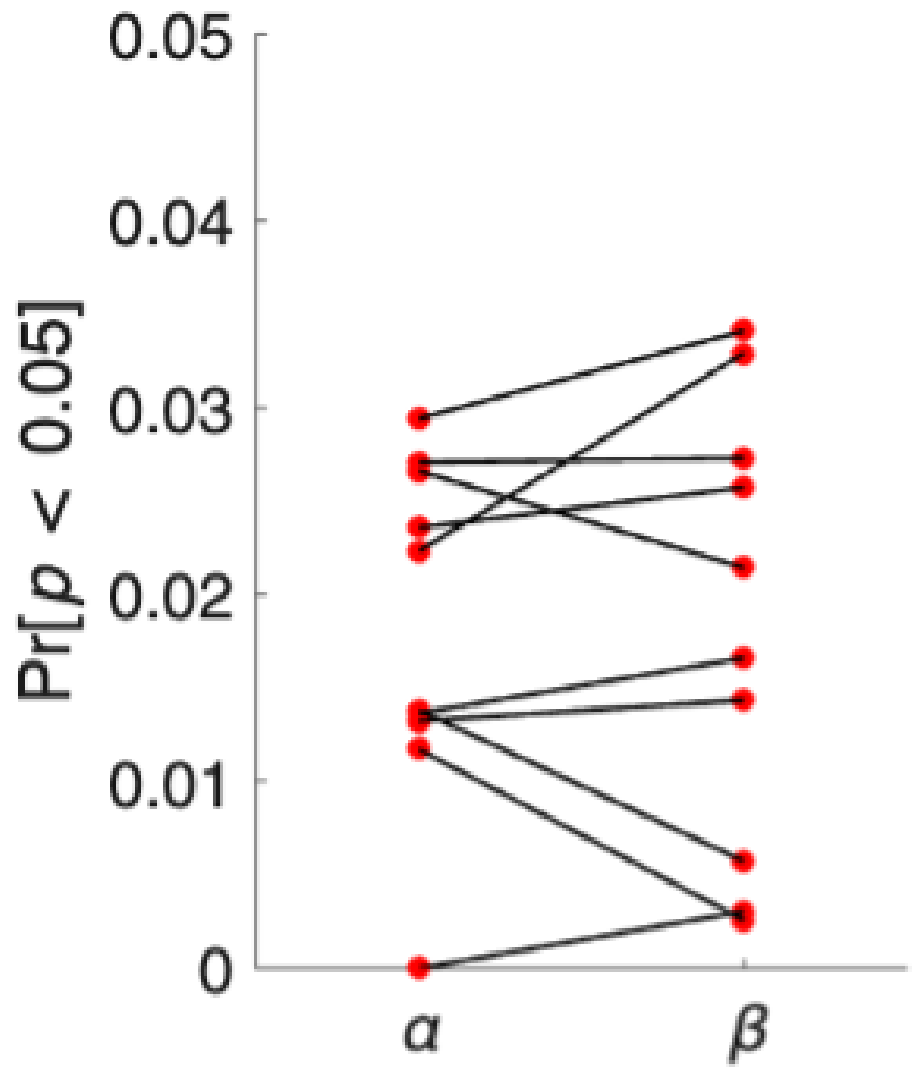

Figure S4. Verification of the statistical test for identifying significant expansion. The statistical test used to identify significant expansion between two time points (Eq. 7, main text) was applied to all sequences with non-zero counts on both day -1 and day 1, such that any expansion detected would constitute a false positive. For both α- and β-chain counts, all individuals analyzed exhibit false-positive rates below 0.05. Data points from the same individual are connected by lines.

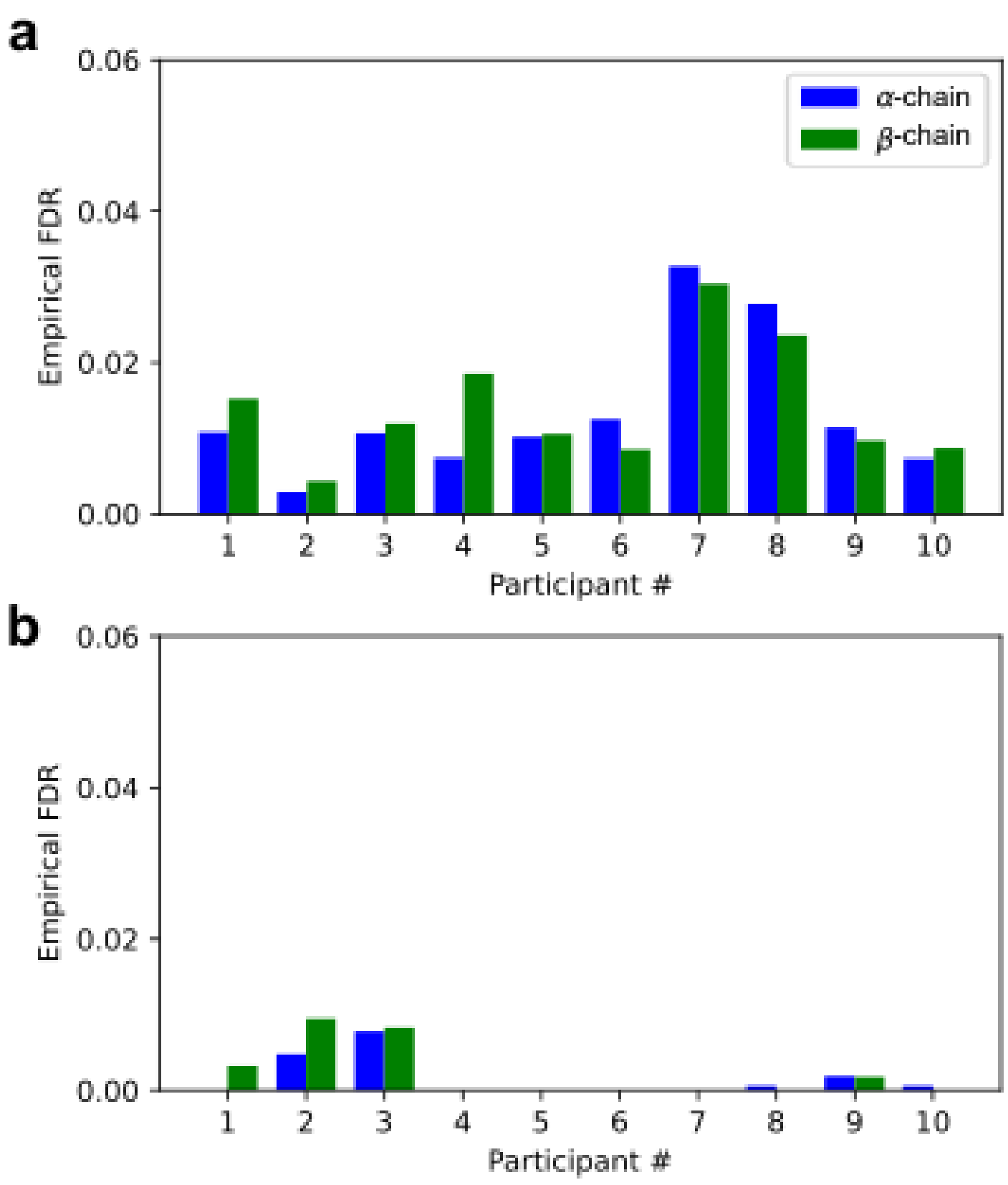

Figure S5. Empirical false discovery rates (FDR) from null estimates. (a) Empirical FDR, defined as the number of clones classified as expanded in the null data divided by the number classified as expanded in the real data, for the case where timepoints were globally reshuffled to destroy temporal correlations. Average FDRs over fifty independent reshufflings are shown. This procedure preserves the per-day clone-size distributions and total sequencing counts while eliminating coherent temporal expansion dynamics and any consistent rise-and-fall structure across timepoints. **(b)** Empirical FDR estimated from a local noise null model constructed from fluctuations between days -1 and 1, which are expected to primarily capture sampling and measurement noise rather than biologically meaningful expansion. Fifteen independent simulations were performed. Simulated trajectories were generated by first sampling a baseline abundance from the empirical day -1 abundance distribution and then independently sampling abundance fluctuations conditioned on that baseline abundance using the empirical day -1 to day 1 fluctuation statistics.

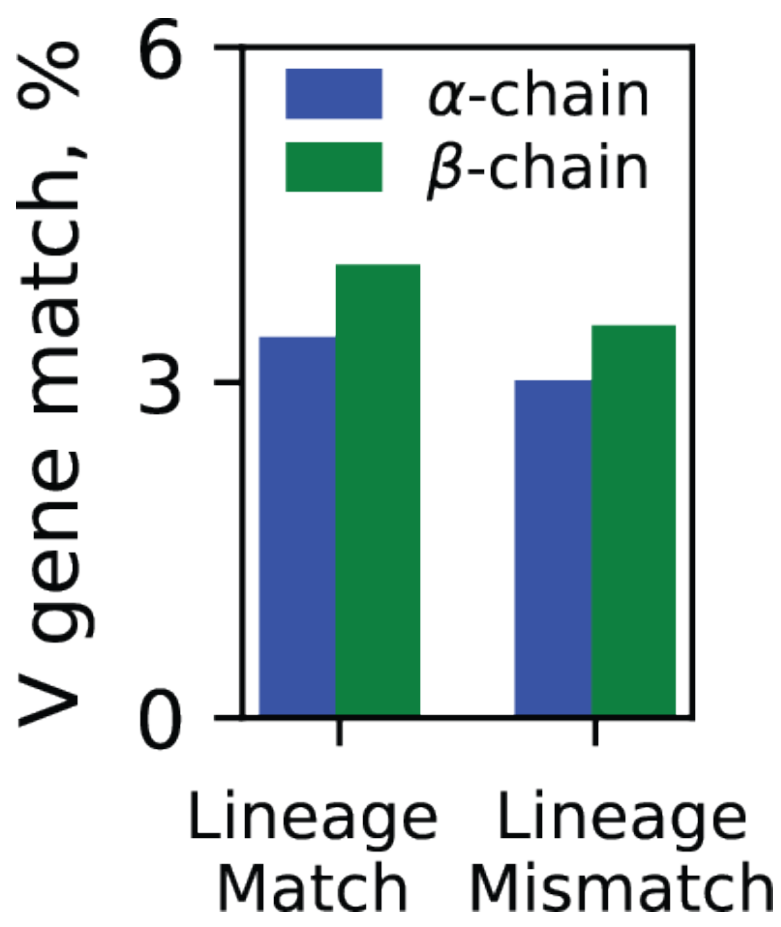

Figure S6. Percentage of unique TCR clone pairs. Unique TCR clone pairs within each individual that shared the same V gene, conditioned on whether the pair was lineage-matched (both $CD4^+$ or both $CD8^+$) or lineage-unmatched (one $CD4^+$, one $CD8^+$) are shown.

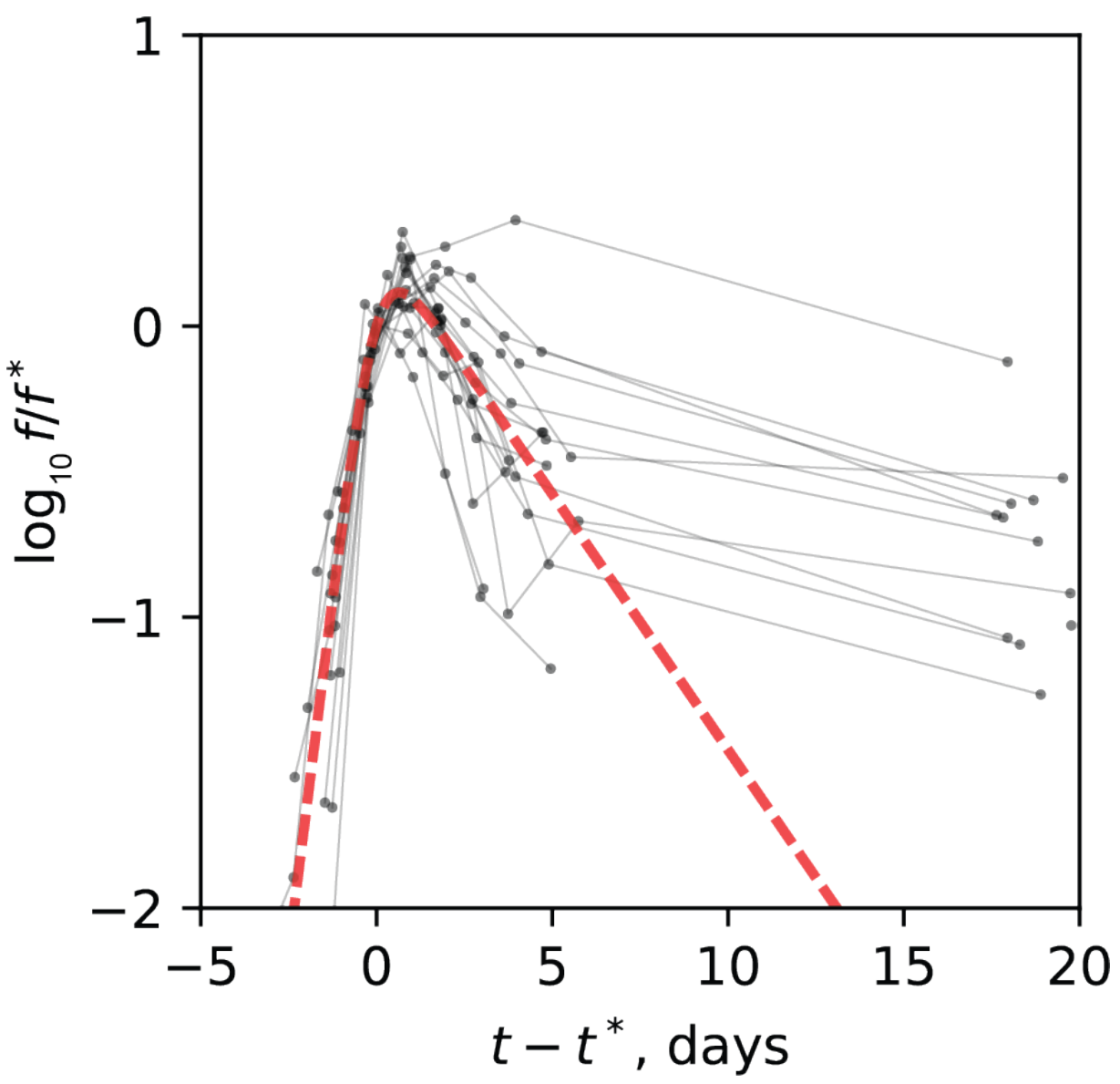

Figure S7. Secondary contraction timescale. Example longitudinal clonal abundance trajectories are shown in black. The red line represents the average fitted trajectory around the peak to guide the eye.

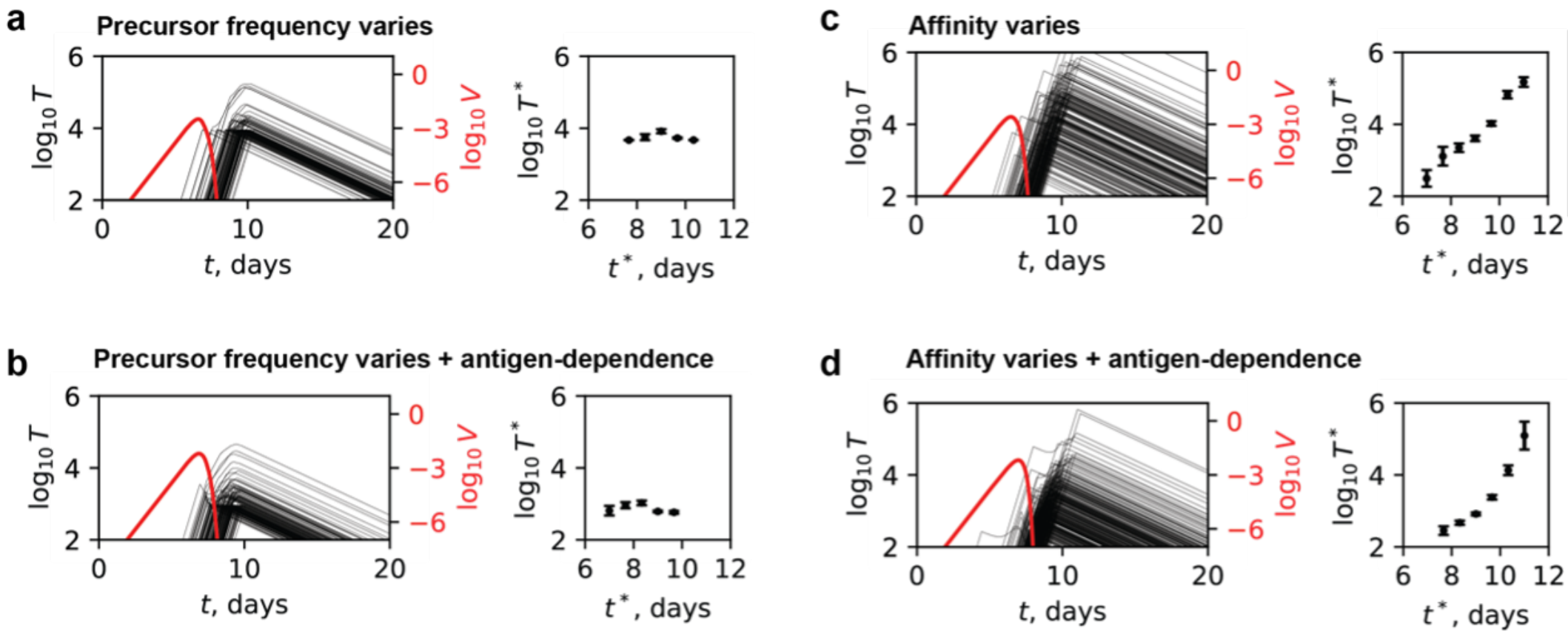

Figure S8. Mathematical model of T cell clonal dynamics with and without antigen-dependent proliferation. Simulated trajectories of antigen-specific T cell clones (black) and viral load (red) are shown in the left panels; corresponding peak T cell abundances, binned by peak day, are shown in the right panels. **a,b** Clones vary in precursor frequency, modeled as a power-law distribution $\Pr[N] \sim N^{-2.2}$, with minimum clone size $N_{\min} = 1.8$ and maximum clone size $N_{\max} = 10^4$, chosen to yield a mean clone size of 10. Results are shown for the division-destiny model (**a**) and for proliferation modulated by antigen availability (**b**). **c,d** Clones vary in affinity, parameterized as $1/K_i = 10^{-5}(1 + 0.2\,\xi_i)$, where $\xi_i \sim \mathcal{N}(0, 1)$. Results are shown for the division-destiny model (**c**) and for antigen-modulated proliferation (**d**).

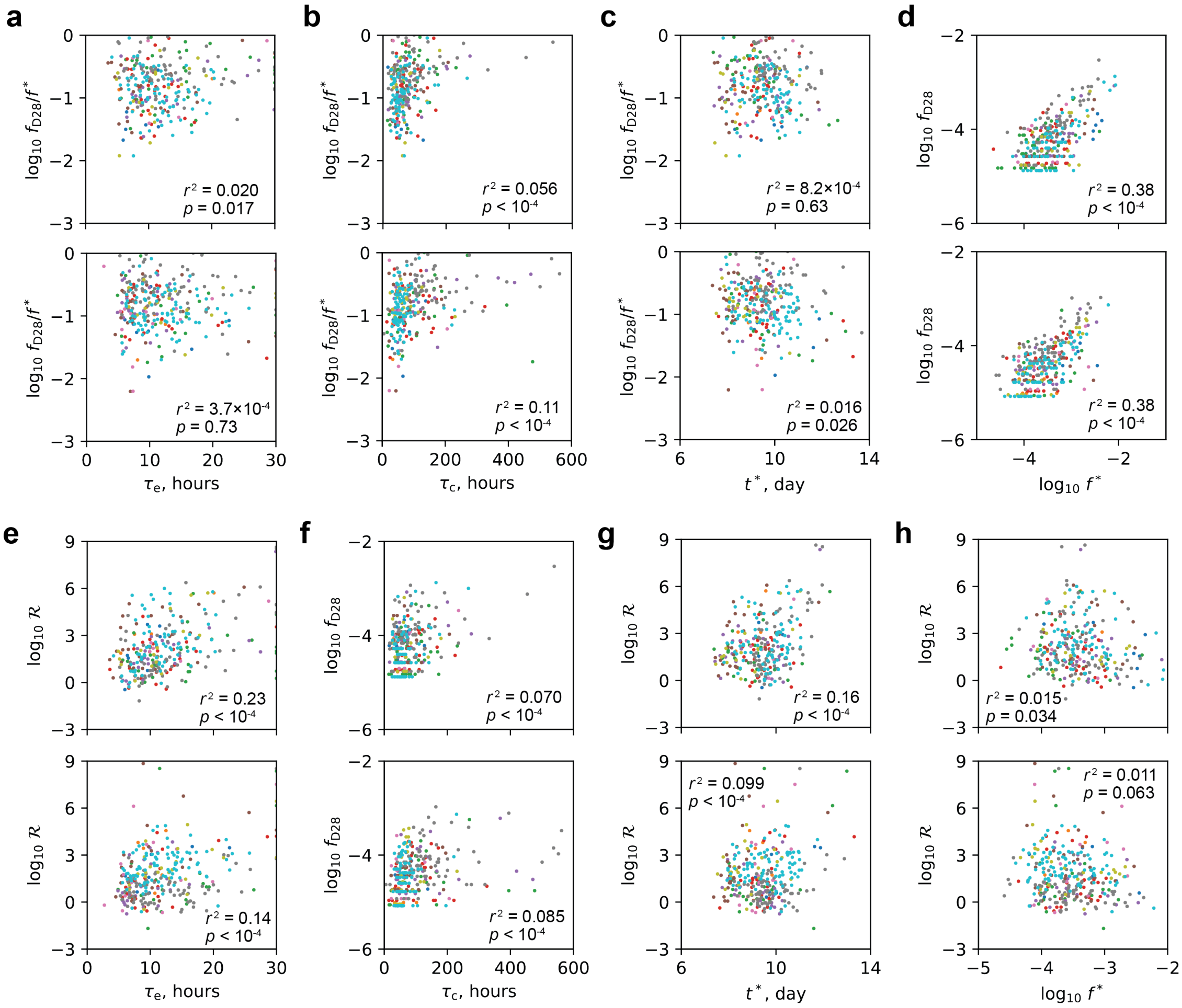

Figure S9. Correlation between clonal persistence and kinetic parameters. a–d, Relationship between clonal persistence, quantified as $\log_{10}(f_{\text{D28}}/f^*)$, and early kinetic parameters: clonal expansion timescale (**a**), contraction timescale (**b**), peak day (**c**), and peak abundance (**d**). **e–h**, Relationship between the deviation from model-predicted abundance, quantified as $\log_{10}\mathcal{R}$, and the same kinetic parameters: clonal expansion timescale (**e**), contraction timescale (**f**), peak day (**g**), and peak abundance (**h**). Correlations were assessed using Pearson's correlation test. Upper panels show measurements based on TCR α-chains, and lower panels show β-chains. Different colors denote data from different individuals. In panels **d** and **f**, $\log_{10} f_{\text{D28}}$ was used in place of $\log_{10}(f_{\text{D28}}/f^*)$ and $\log_{10} R$, respectively, because $\log_{10}(f_{\text{D28}}/f^*)$ is directly influenced by f^* and $\log_{10}\mathcal{R}$ is strongly influenced by τ_{e} in its computation.

REFERENCES

1. S. J. Turner, N. L. La Gruta, K. Kedzierska, P. G. Thomas, P. C. Doherty, Functional implications of T cell receptor diversity. *Curr. Opin. Immunol.* **21**, 286–290 (2009).

2. Q. Qi, Y. Liu, Y. Cheng, J. Glanville, D. Zhang, J.-Y. Lee, R. A. Olshen, C. M. Weyand, S. D. Boyd, J. J. Goronzy, Diversity and clonal selection in the human T-cell repertoire. *Proc. Natl. Acad. Sci. U.S.A.* **111**, 13139–13144 (2014).

3. S. Heinzel, T. Binh Giang, A. Kan, J. M. Marchingo, B. K. Lye, L. M. Corcoran, P. D. Hodgkin, A Myc-dependent division timer complements a cell-death timer to regulate T cell and B cell responses. *Nat. Immunol.* **18**, 96–103 (2017).

4. J. M. Marchingo, G. Prevedello, A. Kan, S. Heinzel, P. D. Hodgkin, K. R. Duffy, T-cell stimuli independently sum to regulate an inherited clonal division fate. *Nat. Commun.* **7**, 13540 (2016).

5. A. Mayer, Y. Zhang, A. S. Perelson, N. S. Wingreen, Regulation of T cell expansion by antigen presentation dynamics. *Proc. Natl. Acad. Sci. U.S.A.* **116**, 5914–5919 (2019).

6. M. K. Jenkins, J. J. Moon, The role of naive T cell precursor frequency and recruitment in dictating immune response magnitude. *J. Immunol.* **188**, 4135–4140 (2012).

7. D. L. Farber, N. A. Yudanin, N. P. Restifo, Human memory T cells: Generation, compartmentalization and homeostasis. *Nat. Rev. Immunol.* **14**, 24–35 (2014).

8. M. Shugay, D. V. Bagaev, I. V. Zvyagin, R. M. Vroomans, J. C. Crawford, G. Dolton, E. A. Komech, A. L. Sycheva, A. E. Koneva, E. S. Egorov, A. V. Eliseev, E. Van Dyk, P. Dash, M. Attaf, C. Rius, K. Ladell, J. E. McLaren, K. K. Matthews, E. B. Clemens, D. C. Douek, F. Luciani, D. van Baarle, K. Kedzierska, C. Kesmir, P. G. Thomas, D. A. Price, A. K. Sewell, D. M. Chudakov, VDJdb: A curated database of T-cell receptor sequences with known antigen specificity. *Nucleic Acids Res.* **46**, D419–D427 (2018).

9. A. A. Minervina, M. V. Pogorelyy, E. A. Komech, V. K. Karnaukhov, P. Bacher, E. Rosati, A. Franke, D. M. Chudakov, I. Z. Mamedov, Y. B. Lebedev, T. Mora, A. M. Walczak, Primary and

secondary anti-viral response captured by the dynamics and phenotype of individual T cell clones. *eLife* **9**, e53704 (2020).

10. R. J. De Boer, D. Homann, A. S. Perelson, Different dynamics of $CD4^+$ and $CD8^+$ T cell responses during and after acute lymphocytic choriomeningitis virus infection. *J. Immunol.* **171**, 3928–3935 (2003).

11. B. Killingley, A. J. Mann, M. Kalinova, A. Boyers, N. Goonawardane, J. Zhou, K. Lindsell, S. S. Hare, J. Brown, R. Frise, E. Smith, C. Hopkins, N. Noulin, B. Löndt, T. Wilkinson, S. Harden, H. McShane, M. Baillet, A. Gilbert, M. Jacobs, C. Charman, P. Mande, J. S. Nguyen-Van-Tam, M. G. Semple, R. C. Read, N. M. Ferguson, P. J. Openshaw, G. Rapeport, W. S. Barclay, A. P. Catchpole, C. Chiu, Safety, tolerability and viral kinetics during SARS-CoV-2 human challenge in young adults. *Nat. Med.* **28**, 1031–1041 (2022).

12. R. G. H. Lindeboom, K. B. Worlock, L. M. Dratva, M. Yoshida, D. Scobie, H. R. Wagstaffe, L. Richardson, A. Wilbrey-Clark, J. L. Barnes, L. Kretschmer, K. Polanski, J. Allen-Hyttinen, P. Mehta, D. Sumanaweera, J. M. Boccacino, W. Sungnak, R. Elmentaite, N. Huang, L. Mamanova, R. Kapuge, L. Bolt, E. Prigmore, B. Killingley, M. Kalinova, M. Mayer, A. Boyers, A. Mann, L. Swadling, M. N. J. Woodall, S. Ellis, C. M. Smith, V. H. Teixeira, S. M. Janes, R. C. Chambers, M. Haniffa, A. Catchpole, R. Heyderman, M. Noursadeghi, B. Chain, A. Mayer, K. B. Meyer, C. Chiu, M. Z. Nikolić, S. A. Teichmann, Human SARS-CoV-2 challenge uncovers local and systemic response dynamics. *Nature* **631**, 189–198 (2024).

13. R. J. De Boer, A. S. Perelson, Towards a general function describing t cell proliferation. *J. Theor. Biol.* **175**, 567–576 (1995).

14. J. M. Marchingo, A. Kan, R. M. Sutherland, K. R. Duffy, C. J. Wellard, G. T. Belz, A. M. Lew, M. R. Dowling, S. Heinzel, P. D. Hodgkin, Antigen affinity, costimulation, and cytokine inputs sum linearly to amplify T cell expansion. *Science* **346**, 1123–1127 (2014).

15. A. J. N. Anelone, S. K. Spurgeon, Modelling and simulation of the dynamics of the antigen-specific T cell response using variable structure control theory. *PLOS ONE* **11**, e0166163 (2016).

16. V. V. Ganusov, D. L. Barber, R. J. De Boer, Killing of targets by CD8+ T cells in the mouse spleen follows the law of mass action. *PLOS ONE* **6**, e15959 (2011).

17. M. U. Gaimann, M. Nguyen, J. Desponds, A. Mayer, Early life imprints the hierarchy of T cell clone sizes. *eLife* **9**, e61639 (2020).

18. P. C. De Greef, S. N. Njeru, C. Benz, S. Fillatreau, B. Malissen, F. Agenès, R. J. De Boer, J. Kirberg, The TCR assigns naive T cells to a preferred lymph node. *Sci. Adv.* **10**, eadl0796 (2024).

19. R. Parsa, H. Assis, T. B. R. De Castro, G. L. Dos Reis, H. Hartweger, A. Bilate, D. Mucida, Tissue-specific clonal selection and differentiation of CD4+ T cells during infection. *Nat. Immunol.* **27**, 725–737 (2026).

20. W. Pan, H. Suami, R. J. Corlett, M. W. Ashton, Lymphatic drainage of the nasal fossae and nasopharynx: Preliminary anatomical and radiological study with clinical implications. *Head Neck* **31**, 52–57 (2009).

21. N. Meegalla, B. W. Downs, "Anatomy, head and neck, palatine tonsil (faucial tonsils)" in *StatPearls* (StatPearls Publishing, Treasure Island (FL), 2025); www.ncbi.nlm.nih.gov/books/NBK538296/.

22. D. Homann, L. Teyton, M. B. A. Oldstone, Differential regulation of antiviral T-cell immunity results in stable CD8+ but declining CD4+ T-cell memory. *Nat. Med.* **7**, 913–919 (2001).

23. M. Milighetti, Y. Peng, C. Tan, M. Mark, G. Nageswaran, S. Byrne, T. Ronel, T. Peacock, A. Mayer, A. Chandran, J. Rosenheim, M. Whelan, X. Yao, G. Liu, S. L. Felce, T. Dong, A. J. Mentzer, J. C. Knight, F. Balloux, E. Greenstein, S. Reich-Zeliger, C. Pade, J. M. Gibbons, A. Semper, T. Brooks, A. Otter, D. M. Altmann, R. J. Boyton, M. K. Maini, A. McKnight, C. Manisty, T. A. Treibel, J. C. Moon, M. Noursadeghi, B. Chain, Large clones of pre-existing T cells drive early immunity against SARS-COV-2 and LCMV infection. *iScience* **26**, 106937 (2023).

24. P. C. De Greef, T. Oakes, B. Gerritsen, M. Ismail, J. M. Heather, R. Hermsen, B. Chain, R. J. De Boer, The naive T-cell receptor repertoire has an extremely broad distribution of clone sizes. *eLife* **9**, e49900 (2020).

25. M. Bajénoff, O. Wurtz, S. Guerder, Repeated antigen exposure is necessary for the differentiation, but not the initial proliferation, of naive $CD4^+$ T cells. *J. Immunol.* **168**, 1723–1729 (2002).

26. W. T. Lee, G. Pasos, L. Cecchini, J. N. Mittler, Continued antigen stimulation is not required during $CD4^+$ T cell clonal expansion. *J. Immunol.* **168**, 1682–1689 (2002).

27. A. G. Schrum, E. Palmer, L. A. Turka, Distinct temporal programming of naive $CD4^+$ T cells for cell division versus TCR-dependent death susceptibility by antigen-presenting macrophages. *Eur. J. Immunol.* **35**, 449–459 (2005).

28. R. Obst, H.-M. Van Santen, D. Mathis, C. Benoist, Antigen persistence is required throughout the expansion phase of a $CD4^+$ T cell response. *J. Exp. Med.* **201**, 1555–1565 (2005).

29. S. Heinzel, J. M. Marchingo, M. B. Horton, P. D. Hodgkin, The regulation of lymphocyte activation and proliferation. *Curr. Opin. Immunol.* **51**, 32–38 (2018).

30. D. A. Blair, D. L. Turner, T. O. Bose, Q.-M. Pham, K. R. Bouchard, K. J. Williams, J. P. McAleer, L. S. Cauley, A. T. Vella, L. Lefrançois, Duration of antigen availability influences the expansion and memory differentiation of T cells. *J. Immunnol.* **187**, 2310–2321 (2011).

31. V. P. Badovinac, B. B. Porter, J. T. Harty, $CD8^+$ T cell contraction is controlled by early inflammation. *Nat. Immunol.* **5**, 809–817 (2004).

32. S. M. Kaech, E. J. Wherry, R. Ahmed, Effector and memory T-cell differentiation: Implications for vaccine development. *Nat. Rev. Immunol.* **2**, 251–262 (2002).

33. S. C. Eisenbarth, Dendritic cell subsets in T cell programming: location dictates function. *Nat. Rev. Immunol.* **19**, 89–103 (2019).

34. T. Oakes, J. M. Heather, K. Best, R. Byng-Maddick, C. Husovsky, M. Ismail, K. Joshi, G. Maxwell, M. Noursadeghi, N. Riddell, T. Ruehl, C. T. Turner, I. Uddin, B. Chain, Quantitative characterization of the T cell receptor repertoire of naïve and memory subsets using an integrated experimental and computational pipeline which is robust, economical, and versatile. *Front. Immunol.* **8**, 1267 (2017).

35. I. Uddin, K. Joshi, T. Oakes, J. M. Heather, C. Swanton, TRACERx consortium, B. Chain, "An economical, quantitative, and robust protocol for high-throughput T cell receptor sequencing from tumor or blood" in *Methods in Molecular Biology* (Springer New York, New York, NY, 2019), pp. 15–42; http://link.springer.com/10.1007/978-1-4939-8885-3_2.

36. T. Peacock, J. M. Heather, T. Ronel, B. Chain, Decombinator V4: An improved AIRR-C compliant-software package for T-cell receptor sequence annotation? *Bioinformatics* **37**, 876–878 (2021).

37. D. L. Chao, M. P. Davenport, S. Forrest, A. S. Perelson, A stochastic model of cytotoxic T cell responses. *J. Theor. Biol.* **228**, 227–240 (2004).

38. J. A. M. Borghans, R. J. De Boer, L. A. Segel, Extending the quasi-steady state approximation by changing variables. *Bull. Math. Biol.* **58**, 43–63 (1996).

39. I. Bains, R. Antia, R. Callard, A. J. Yates, Quantifying the development of the peripheral naive $CD4^+$ T-cell pool in humans. *Blood* **113**, 5480–5487 (2009).

40. M. F. Krummel, F. Bartumeus, A. Gérard, T cell migration, search strategies and mechanisms. *Nat. Rev. Immunol.* **16**, 193–201 (2016).

41. T. P. Arstila, A. Casrouge, V. Baron, J. Even, J. Kanellopoulos, P. Kourilsky, A direct estimate of the human αβ T cell receptor diversity. *Science* **286**, 958–961 (1999).

42. J. N. Mandl, R. Liou, F. Klauschen, N. Vrisekoop, J. P. Monteiro, A. J. Yates, A. Y. Huang, R. N. Germain, Quantification of lymph node transit times reveals differences in antigen surveillance strategies of naïve $CD4^+$ and $CD8^+$ T cells. *Proc. Natl. Acad. Sci. U.S.A.* **109**, 18036–18041 (2012).
